# Ekbom syndrome - a case report

**DOI:** 10.1192/j.eurpsy.2021.1437

**Published:** 2021-08-13

**Authors:** S. Nascimento, H. Simião, T. Mendonça, M. Silva

**Affiliations:** 1 Psychiatry, Centro hospitalar psiquiátrico de lisboa, Lisboa, Portugal; 2 Psychiatry, Centro Hospitalar de Lisboa Ocidental, Lisbon, Portugal; 3 Psychiatry, Hospital Garcia de Orta, Almada, Portugal

**Keywords:** Delusional parasitosis, delusional infestation, Ekbom syndrome

## Abstract

**Introduction:**

Delusional parasitosis/infestation or Ekbom syndrome is an uncommon psychotic disorder characterized by a false belief that there is a parasitic infestation of the skin - the delusion that insects are crawling underneath the skin.

**Objectives:**

This work aims to summarize and evaluate the currently available evidence regarding Delusional parasitosis, and for this purpose, we will illustrate a case report of a patient admitted in the emergency room.

**Methods:**

The authors have conducted online research in PubMed with the words “Delusional parasitosis” “delusional infestation”, “Ekbom syndrome”, from the outcome, the articles considered to be relevant were collected and analyzed.

**Results:**

Delusional parasitosis can be classified into primary delusional parasitosis without other psychiatric or organic disorders present, secondary – functional (secondary to several mental disorders such as schizophrenia, depression, dementia, anxiety, and phobia), and organic forms (associated with hypothyroidism, anaemia, vitamin B12 deficiency, hepatitis, diabetes, infections (e.g., HIV, syphilis), and cocaine abuse. It is most commonly seen in middle-aged women. The patients became frequently socially isolated, prone to the development of depression symptoms.

**Conclusions:**

This syndrome often presents a high level of psychosocial morbidity. Patients often seek dermatologists help in the first place, although there is no medical evidence. Psychiatrists play a major role in the diagnosis and treatment of these patients. Psychopharmacological therapy is quite challenging because of the patient’s belief that they have a parasitic infestation and not a psychiatric condition.

